# Von Hippel-Lindau Disease: The Importance of Retinal Hemangioblastomas in Diagnosis

**DOI:** 10.4274/tjo.90912

**Published:** 2017-06-01

**Authors:** Sevinç Şahin Atik, Aslı Ece Solmaz, Zafer Öztaş, Emine Deniz Eğrilmez, Şeyda Uğurlu, Tahir Atik, Filiz Afrashi

**Affiliations:** 1 Katip Çelebi University Atatürk Training and Research Hospital, Ophthalmology Clinic, İzmir, Turkey; 2 Ege University Faculty of Medicine, Department of Medical Genetics, İzmir, Turkey; 3 Ege University Faculty of Medicine, Department of Ophthalmology, İzmir, Turkey; 4 Ege University Faculty of Medicine, Department of Pediatrics, İzmir, Turkey

**Keywords:** Renal cell carcinoma, retinal hemangioblastoma, Von Hippel-Lindau syndrome

## Abstract

Von Hippel-Lindau (VHL) disease is a familial cancer syndrome characterized by benign or malignant tumors which may involve more than one system. Retinal hemangioblastomas are usually the initial manifestation of VHL disease and can cause vision loss. A 32-year-old man presented to our clinic with vision loss in the left eye for 2 months. He had a history of cerebral hemangioblastoma operation. Family history showed that his mother had unilateral vision loss and died because of renal cell carcinoma. Ophthalmologic examination revealed multiple retinal hemangioblastomas in both eyes. VHL gene sequencing was performed and heterozygous p.R161X mutation was detected. His sister and daughter were also found to have the same variant. A treatment and follow-up plan was initiated for the patient and affected family members. Considering VHL disease in the differential diagnosis of retinal hemangioblastomas has a very important role in the early detection of life-threatening tumors in these patients.

## INTRODUCTION

Von Hippel-Lindau (VHL) disease a familial cancer syndrome characterized by benign or malignant tumors and cystic lesions affecting multiple systems. It may present with hemangioblastomas in the brain, spinal cord, and retina; renal cysts and clear cell renal cell carcinoma; pheochromocytoma; pancreatic cysts and neuroendocrine tumors; endolymphatic sac tumors; and epididymal and ligamentum latum cysts. Retinal hemangioblastomas are usually the initial sign of VHL disease and can cause vision loss. Up to 70% of affected individuals are diagnosed at an average age of 25 years old by detection of retinal hemangioblastomas.^1,2,3^ Renal cell carcinoma is seen in about 70% of VHL patients and constitutes the greatest cause of mortality.^4^ Identifying patients with retinal hemangioblastoma and evaluating them for VHL is of vital importance to both the patients and their relatives. Herein, we present a case diagnosed with VHL based on retinal hemangioblastomas after presenting to our clinic with reduced vision, and the treatment and follow-up of the patient and his relatives.

## CASE REPORT

**Figure 1:**
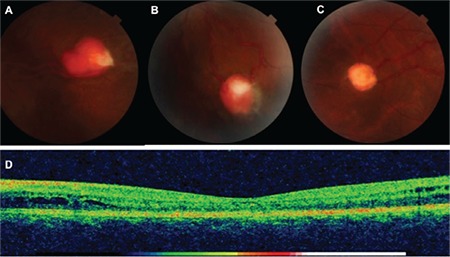
Retinal hemangioblastomas located in the (A) superotemporal left eye, (B) inferotemporal left eye, and (C) peripheral right eye, (D) Ocular computed tomography image showing perifoveal intraretinal cystic fluid accumulation

**Figure 2:**
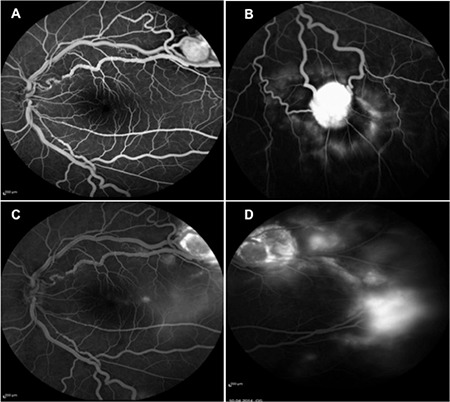
Left eye fundus fluorescein angiography images showing (A) increased arterial and venous diameter and hyperfluorescent appearance of the superotemporal hemangioblastoma, (B) inferotemporal hemangioblastoma, (C) edema in the temporal macula, and (D) increased late fluorescein leakage around the superotemporal hemangioblastoma

A 32-year-old male patient presented to our outpatient clinic with vision loss in his left eye that started 2 months earlier. Ophthalmologic examination revealed no refractive error; visual acuity was 1.0 in the right eye and 0.2 in the left eye. Intraocular pressure was 14 mmHg in both eyes. Slit-lamp examination was normal. Bilateral increases in arterial and venous diameters and increased tortuosity were observed on fundus examination. Retinal hemangioblastomas were observed in the superotemporal (size, 3.63x4.67 mm) and inferotemporal (size, 2.95x3.41 mm) retina of the left eye and in the temporal (size, 2.49x2.54 mm) retina of the right eye (Figure 1a-c). The hemangioblastomas showed early hyperfluorescence on fundus fluorescein angiography (FFA). The feeding arteries and draining veins were clearly visible. Fluorescein leakage was observed around the temporal hemangioblastomas and macular region in the left eye (Figure 2). Optic coherence tomography (OCT) in the left eye revealed intraretinal cystic fluid accumulation in the perifoveal region (Figure 1d).

In his medical history, the patient reported undergoing surgery approximately 8 years earlier due to a cranial mass. He also reported that his mother had suffered from advanced unilateral vision loss and died of renal cancer. In the pathology report from the patient’s brain surgery, the tissue was identified as hemangioblastoma. VHL was suspected based on the presence of multiple, bilateral retinal hemangioblastomas and history of cerebral hemangioblastoma. VHL gene sequence analysis revealed a heterozygous p.R161X mutation.

**Figure 3:**
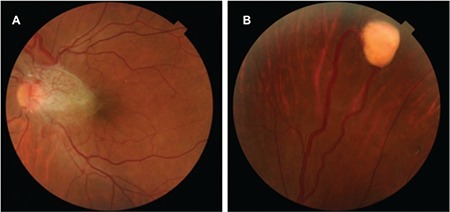
Images from the patient’s sister’s left eye showing (A) a hemangioblastoma on the optic nerve head and (B) a hemangioblastoma in the peripheral temporal retina

**Figure 4:**
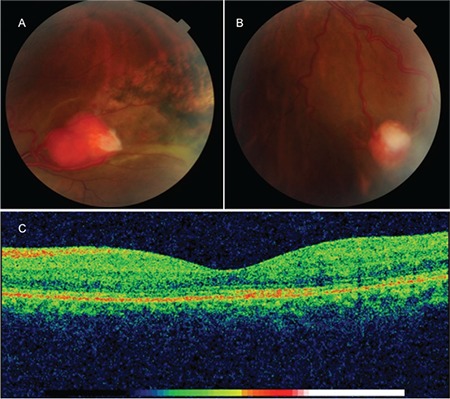
Images of the (A) superotemporal and (B) inferotemporal hemangioblastomas in the left eye after cryotherapy. (C) Ocular computed tomography image showing regression of the perifoveal intraretinal cystic fluid

The patient was diagnosed with VHL and underwent examination and imaging of the other systems. A cortical cyst 13 mm in diameter was detected in the left kidney on renal ultrasonography (USG) and the patient was followed in the department of nephrology with USG examinations at 6-month intervals. Due to the patient’s vision loss and intraretinal cystic fluid collection in the left eye, cryotherapy with three freeze/thaw cycles was applied only to the superotemporal hemangioblastoma. The other lesions were monitored with examination and imaging. A reduction in the perifoveal intraretinal fluid was observed after cryotherapy. On examination 4 months after cryotherapy, visual acuity in the left eye was 0.4 and no perifoveal intraretinal fluid was visible on OCT (Figure 3). The patient’s family members were also invited to the clinic to be evaluated for VHL. Ophthalmologic examination of the patient’s sister revealed visual acuity of 0.2 in the left eye and an optic nerve head hemangioblastoma causing macular traction and edema (Figure 4). Another hemangioblastoma (size, 2.90x2.35 mm) was detected in the peripheral temporal retina of the same eye. She had full vision and fundus examination was normal in the right eye. Examination and imaging of her other systems was performed. Abdominal USG revealed cystic lesions in the pancreas and kidney; advanced study with contrast magnetic resonance imaging was recommended. No pathology was detected in ophthalmologic examination of the patient’s 12-year-old daughter.

Based on these findings, VHL gene analysis was planned for the patient’s sister and daughter. Both were found to be heterozygous for the p.R161X mutation. Intravitreal anti-vascular endothelial growth factor inhibitor therapy was planned for the patient’s sister due to the hemangioblastoma on her optic nerve head. However, it was then determined that she was 6 weeks pregnant. As this agent is category C for use during pregnancy and its absolute benefit is still debated, it was decided to monitor the patient without treatment. She was referred to genetic counseling, and pregnancy follow-up and prenatal diagnosis were planned. All patients were followed for possible complications by the relevant departments.

## DISCUSSION

VHL disease is a rare, autosomal dominant, monogenic disease arising from heterozygous mutations of the VHL gene, located on chromosome 3p25.3. The incidence of VHL has been determined as approximately 1 in 36,000 live births.^5^ Seventy-two percent of patients can be diagnosed with VHL gene sequence analysis; large exonic or whole gene deletions or duplications are responsible for the remaining 28%.^6^ The p.R161X mutation identified in the family in the present study is a nonsense mutation described previously in the literature.^7^ According to the phenotypic characteristics, VHL disease can be divided into 4 phenotypic groups based on the presence of pheochromocytoma or renal cell carcinoma (type 1, type 2A, type 2B, and type 2C). Pheochromocytoma is not present in type 1, but occurs with all other types. Considering his clinical findings and family/medical history, our patient was consistent with type 1. Although further evidence is needed in the literature to establish the genotype/phenotype relationship, it has been reported that missense and nonsense mutations lead to the type 1 VHL phenotype.^8^

Retinal hemangioblastomas are generally located in the peripheral temporal retina, but may rarely occur in the posterior pole and on the optic disc. Lesions are usually bilateral and multiple.^9^ The differential diagnosis should include Coat’s disease, retinal cavernous hemangioma, retinal macroaneurysm, and vasoproliferative tumor.^10^

In the retina, hemangioblastomas are slow-growing, benign tumors. Without treatment, however, they can cause complications such as macular edema, exudative and tractional retinal detachment, intravitreal hemorrhage, and neovascular glaucoma.^11^ There is no standard treatment approach to retinal hemangioblastomas. The treatment method varies depending on the hemangioblastoma’s location, size, and related complications. Treatment options include laser photocoagulation, cryotherapy, photodynamic therapy, transpupillary thermotherapy, plaque radiotherapy, external beam radiotherapy, and vitreoretinal surgical ablation.^12,13,14^ Intravitreal anti-vascular endothelial growth factor inhibitors have recently come into use for reducing exudation resulting from hemangioblastoma.^15^ Active surveillance is recommended for juxtapapillary hemangioblastomas and peripheral hemangioblastomas smaller than 500 microns that do not cause exudation or subretinal fluid. Laser photocoagulation is more common to treat small tumors, while cryotherapy is more often preferred for very peripheral tumors larger than 3 mm.^12^ We chose cryotherapy for our patient because the hemangioblastoma causing perifoveal intraretinal cystic fluid collection in the left eye was larger than 4.5 mm in size and was situated peripherally. Cryotherapy resulted in regression of the perifoveal intraretinal cystic fluid and improved visual acuity. Early diagnosis and appropriate treatment reduces the risk of vision loss, especially with tumors located in the periphery. The likelihood of favorable outcome is lower with tumors located on or around the optic disc.^9^

In this case, VHL disease was suspected based on findings of multiple retinal hemangioblastomas and was confirmed with genetic testing. Although the patient had previously undergone surgery for cerebral hemangioblastoma, it was apparent that the possible role of VHL in its etiology had not been investigated through a comprehensive differential diagnosis. We also informed the patient’s relatives about this genetic condition and performed the necessary examination and tests. Because hemorrhages associated with renal cancer and cerebral hemangioblastomas are the leading causes of mortality in these patients at young ages, systemic evaluation and monitoring is crucial. Our patient and his sister were diagnosed with renal cysts and routine follow-up was scheduled. The patients were advised to avoid tobacco products, as well as chemical and industrial toxins. They were also recommended to avoid contact sports due to their renal and pancreatic lesions.

As an ophthalmologist, identifying retinal hemangioblastomas and determining whether they are related to VHL is extremely important for the early diagnosis and treatment of life-threatening tumors and complications that may develop in these patients and their families.
